# Permanent Draft Genome Sequence for *Frankia* sp*.* Strain EI5c, a Single-Spore Isolate of a Nitrogen-Fixing Actinobacterium, Isolated from the Root Nodules of *Elaeagnus angustifolia*

**DOI:** 10.1128/genomeA.00660-16

**Published:** 2016-07-07

**Authors:** Timothy D’Angelo, Rediet Oshone, Feseha Abebe-Akele, Stephen Simpson, Krystalynne Morris, W. Kelley Thomas, Louis S. Tisa

**Affiliations:** University of New Hampshire, Durham, New Hampshire, USA

## Abstract

*Frankia* sp. strain EI5c is a member of *Frankia* lineage III, which is able to reinfect plants of the *Eleagnaceae*, *Rhamnaceae*, *Myricaceae*, and *Gymnostoma*, as well as the genus *Alnus*. Here, we report the 6.6-Mbp draft genome sequence of *Frankia* sp. strain EI5c with a G+C content of 72.14 % and 5,458 candidate protein-encoding genes.

## GENOME ANNOUNCEMENT

Actinorhizal plants form a nitrogen-fixing symbiosis with the genus *Frankia* that results in the ability of these plants to colonize harsh environments (1–3). These important pioneer community plants are found worldwide under a wide range of ecological and environmental conditions, especially in poor and marginal fertile soils (3,4). Actinorhizal plants represent 8 different families of angiosperms and over 200 species of woody dicotyledonous plants.

Based on phylogenetic markers, four major clusters are recognized within the genus (5–8). Cluster I consists of *Frankia* strains that associate with host plants in the *Casuarinaceae*, *Betulaceae*, and *Myricaceae* families, while members of cluster II are infective on *Rosaceae, Coriariaceae, Datiscaceae*, and the genus *Ceonothus* (*Rhamnaceae*). Cluster III members are the most promiscuous and are infective on *Eleagnaceae*, *Rhamnaceae*, *Myricaceae*, *Gymnostoma*, and occasionally the genus *Alnus*. Cluster IV consists of “atypical” *Frankia* strains that are unable to reinfect actinorhizal host plants or form ineffective root nodule structures that are unable to fix nitrogen. Genomes for representatives from each cluster have been sequenced (9–25) and have provided a rich database. Analysis of that resource has revealed several physiological properties, including metabolic diversity, natural product biosynthesis, and stress tolerance.

Besides being broad-host-range symbionts, members of cluster III have the greatest metabolic diversity and possess larger genomes than the other clusters. Many of these strains have adapted to harsh environmental conditions. *Frankia* sp. strain EI5c was obtained from a single-spore colony isolation from cultures of *Frankia* sp. strain EI5 (UFI 132715) (26), which was isolated from root nodules of *Elaeagnus angustifolia* (27). This strain uses both intracellular (root hair infection) and intercellular pathways of infection. *Frankia* sp. strain EI5c was sequenced to provide information on this lineage and its interactions with actinorhizal plants. Furthermore, this database will also be used to help clarify the diversity of cluster III members with the goal of speciation.

The draft genomes of *Frankia* sp. strain EI5c were generated at the Hubbard Genome Center (University of New Hampshire, Durham, NH, USA) using Illumina technology (28) techniques. A standard Illumina shotgun library was constructed and sequenced using the Illumina HiSeq2000 platform, which generated 24,085,096 reads (260-bp insert size) totaling 3,612.7 Mbp. The Illumina sequence data were assembled using CLC Genomics workbench version 8.0.1 and AllPaths-LG version r41043 (29). The final draft assembly for *Frankia* EI5c consisted of 159 contigs with an *N*_50_ contig size of 128.2 kb. The final assembled genome contained a total sequence of 6,617,243 bp with a G+C content of 72.14 % and is based on 1,290.3 Mb of Illumina draft data, providing an average 195× coverage of the genome.

The assembled *Frankia* sp. strain EI5c genome was annotated via the Integrated Microbial Genomes (IMG) platform developed by the Joint Genome Institute, Walnut Creek, CA, USA (30, 31), and resulted in 5,458 candidate protein-encoding genes and 46 tRNA and 2 rRNA regions.

### Nucleotide sequence accession numbers.

This whole-genome shotgun project has been deposited at DDBJ/EMBL/GenBank under the accession number LRTK00000000. The version described in this paper is the first version, LRTK01000000.
